# Scaling and mechanical optimality of bristled wings in microinsects

**DOI:** 10.1073/pnas.2506403122

**Published:** 2025-08-22

**Authors:** Dmitry Kolomenskiy, Sergey E. Farisenkov, Pyotr N. Petrov, Alexey A. Polilov

**Affiliations:** ^a^Center for Materials Technologies, Skolkovo Institute of Science and Technology, Moscow 121205, Russia; ^b^Department of Entomology, Faculty of Biology, Lomonosov Moscow State University, Moscow 119234, Russia; ^c^Joint Russian-Vietnamese Tropical Research and Technological Center, Ho Chi Minh City 70000, Vietnam

**Keywords:** animal flight, allometry, miniaturization

## Abstract

It is crucial for both animal evolution and engineering to optimize the relative size of structures. Animal wings are no exception, every structural design having its limits in terms of achievable size and performance. For instance, many microinsects have bristled wings, which are more efficient at small scales than the membranous wings common in larger insects. However, the limitations and the optimal characteristics of bristled wings remain largely underinvestigated. We collected morphological and kinematic data on a variety of beetles ranging between 0.3 and 5 mm in wing length. This was followed by a theoretical analysis to explain from the mechanical standpoint the morphological traits and allometric scalings observed in the data. We derived functional dependencies for parameters such as the number of bristles, bristle length and diameter, size of the wing blade, etc., from considerations of wing inertia minimization under the aerodynamic and structural stiffness constraints. The solution of the optimization problem reveals scaling relationships aligning with empirical trends, which suggests that the reduction of wing membrane during miniaturization can be explained by mechanical optimality. Thus, scaling of the number of bristles and the average gap width between bristles follows directly from the aerodynamic condition of maintaining low permeability, while the bristle diameter and length are determined mainly by the structural stiffness requirement. Similar mechanical arguments are likely applicable to other miniature animals that propel through fluids.

## Significance

The bristled wings of miniature insects suggest an evolutionary advantage over membranous wings at small sizes, yet a quantitative understanding of this advantage has been limited due to the lack of comparative biomechanical studies. Our work addresses this gap by deriving a physically based mathematical model and using statistical regression on empirical data acquired by light and scanning electron microscopy and by high-speed videography, to define scaling laws and identify size limits for optimal performance of bristled wings. These results highlight the determining role of mechanical factors in evolutionary adaptations of microinsects, offering quantitative insights into why these animals are able to thrive in their ecological niches. They can serve as models for efficient design of future ultraminiature flapping-wing vehicles.

**Fig. 1.**
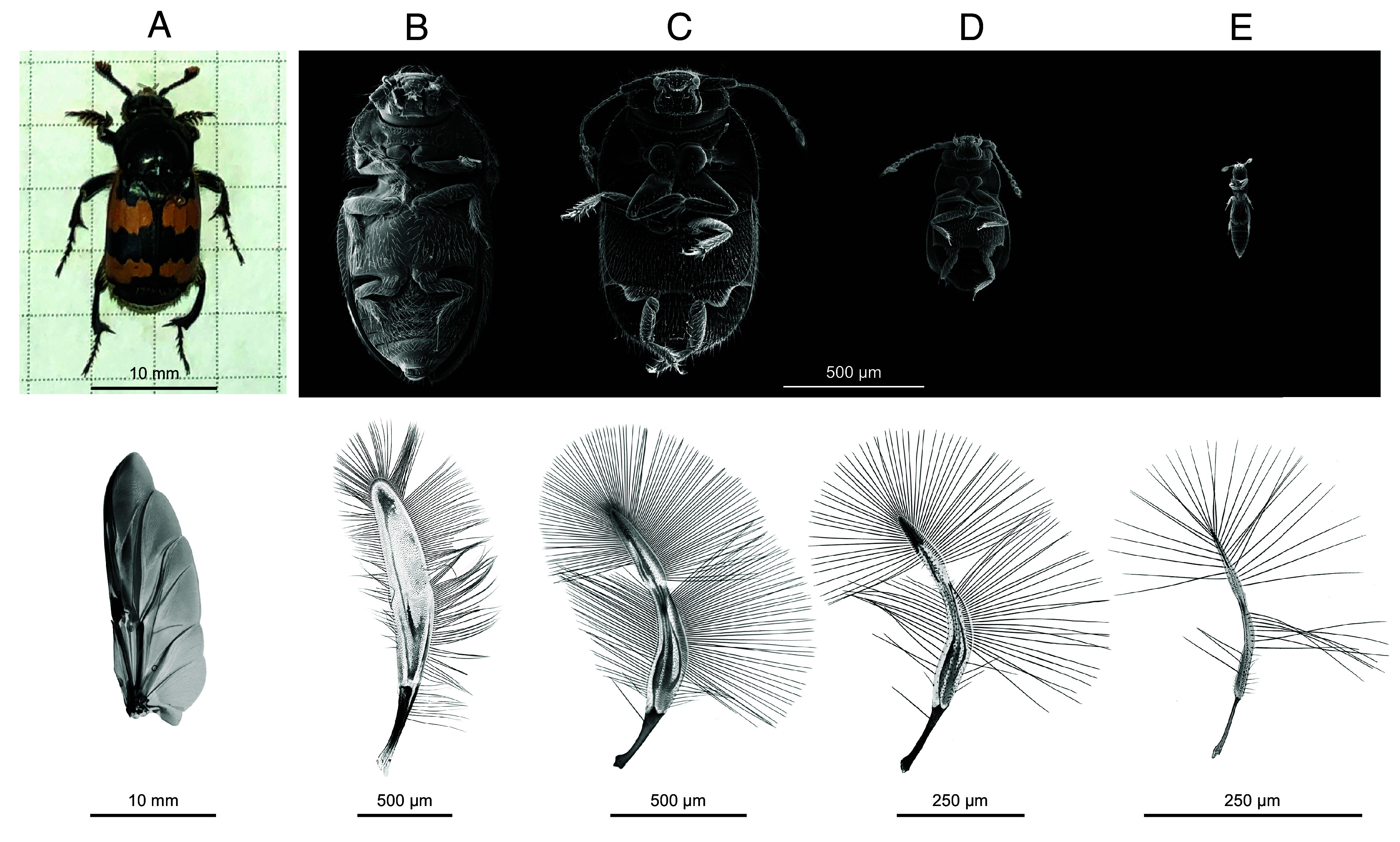
Habitus and wings of beetles in the body size range from 21 mm down to 0.34 mm. A membranous-winged burying beetle (Staphylinidae: Silphinae) *Nicrophorus vespillo* (*A*) in comparison with smaller featherwing beetles (Ptiliidae), in the order of decreasing size: *Sindosium* sp. (*B*); *Acrotrichis grandicollis* (*C*); *Nephanes titan* (*D*); and *Scydosella musawasensis* (*E*).

Insects vary in size vastly, yet the majority can fly by flapping their wings. The wing lengths range from a fraction of a millimeter in miniature species (such as featherwing beetles considered in this study; see Fig. 1*B*–*E*) to over 10 cm in the largest insects (e.g., 15 cm in *Thysania agrippina* moths). The majority of species larger than 2 mm long have membranous wings that consist of thin impermeable membrane cells tense between thicker veins, which provide the necessary bending stiffness (Fig. 1*A*). Their aerodynamic and structural properties have been a focus of research for several decades. But wings of the smallest insects are strikingly dissimilar morphologically: the number of veins is drastically reduced, the membrane covers only a small region, and a fringe of long bristles extends on the periphery (Fig. 1*B*–*E*, lower row). This feather-like shape, termed ptyloptery as a morphological trait (1), was described by entomologists more than a century ago, but its mechanical function remained elusive until the advent of suitable measurement tools such as scanning electron microscopy for the morphological measurement and synchronized high-speed videography for three-dimensional kinematic reconstruction.

Miniature insects showcase exotic wing motions with large out-of-plane deviations and angles of attack, in contrast to larger insects (2). These kinematic features are shared among insects with varying degrees of ptyloptery and the smallest species with purely membranous wings. Concurrent aerodynamic analyses (as reviewed in ref. 3) provide ample evidence that minute bristled wings generate aerodynamic forces of the same order of magnitude as similarly sized membranous ones. For instance, the featherwing beetle *Paratuposa placentis* develops 70% of the aerodynamic force despite over 95% reduction of the membrane area, relative to a hypothetical membranous wing of the same outline (4). This is possible because viscous stresses efficiently block the air flow through gaps between the bristles, making the membrane unnecessary from the aerodynamic point of view. To characterize this effect, Lee et al. (5) suggested to use the gap-based Reynolds number $Re_{G}=UG/\nu$, where U is the characteristic velocity, G is the characteristic gap width between bristles, and ν is the kinematic viscosity of air. At high $Re_{G}$, individual bristles function as isolated cylindrical rods, whereas in the low $Re_{G}$ limit, the aerodynamic interference grows so that the total force converges to that of an impermeable wing. The parameter $Re_{G}$ can be evaluated based on the published data to fall in the range between 0.1 and 1 (6), but in the present study we focus on featherwing beetles (Ptiliidae) to make a sharper estimate.

Reduction of the wing membrane helps to reduce the inertia and, consequently, minimize the loads on flight muscles (4). However, in addition to impermeability, the membrane also contributes to structural stiffness. Individual bristles without tensile support must be thick enough to resist flexing forces. The available data show that the bristles are indeed sufficiently stiff to prevent accidental bending reconfiguration and the concomitant loss of aerodynamic force (7). These considerations prompt us to regard the ptyloptery condition as a constrained optimization problem. The wing moment of inertia is to be minimized under the constraints of necessary aerodynamic force and structural stiffness. The optimal parameters will presumably depend on the size, and morphological observations on Ptiliidae (8) show that the bristle diameter and spacing vary allometrically.

In the present study, we expand further the morphological dataset and supplement it with free flight kinematic measurements in a wider range of beetles of the superfamily Staphylinoidea (which includes Ptiliidae). The quantities included in the new dataset are selected so as to enable the calculation of representative values of $Re_{G}$ and the wing beat frequency f. These two parameters being determined as family-specific, we state the optimization problem for an idealized mathematical model of a beetle avoiding any further need for empirical calibration. We focus on a single insect order, Coleoptera, to minimize interspecific variance and thereby facilitate the application of self-similarity assumptions in the mathematical model. A parameter sweep through a range of values of the wing length produces optimal scaling relationships for the morphological features included in the model, explaining the empirical allometric statistical trends derived directly from the morphological measurement data.

## Results and Discussion

### Radius of the Second Moment of Wing Area.

To perform order-of-magnitude aerodynamic calculations, it is crucial to appropriately define the velocity scale. The wing velocity at the radius of the second moment of wing area is conventionally used in animal flight research, e.g., for determining the Reynolds number (9). This choice of reference velocity ensures that the numerical value of the force coefficient of the entire wing is close to the sectional aerodynamic force coefficient at the distance $R_{2}$ from the wing base (*SI Appendix*, section 3), which makes it best suited for quick aerodynamic force estimates.

For bristled wings, the aerodynamically effective area is much larger than the planar projection area, because flow through gaps between bristles is blocked by viscosity. For this reason, when we calculate $R_{2}$, we treat spaces between bristles as membranous cells, and use the formula

**Fig. 2.**
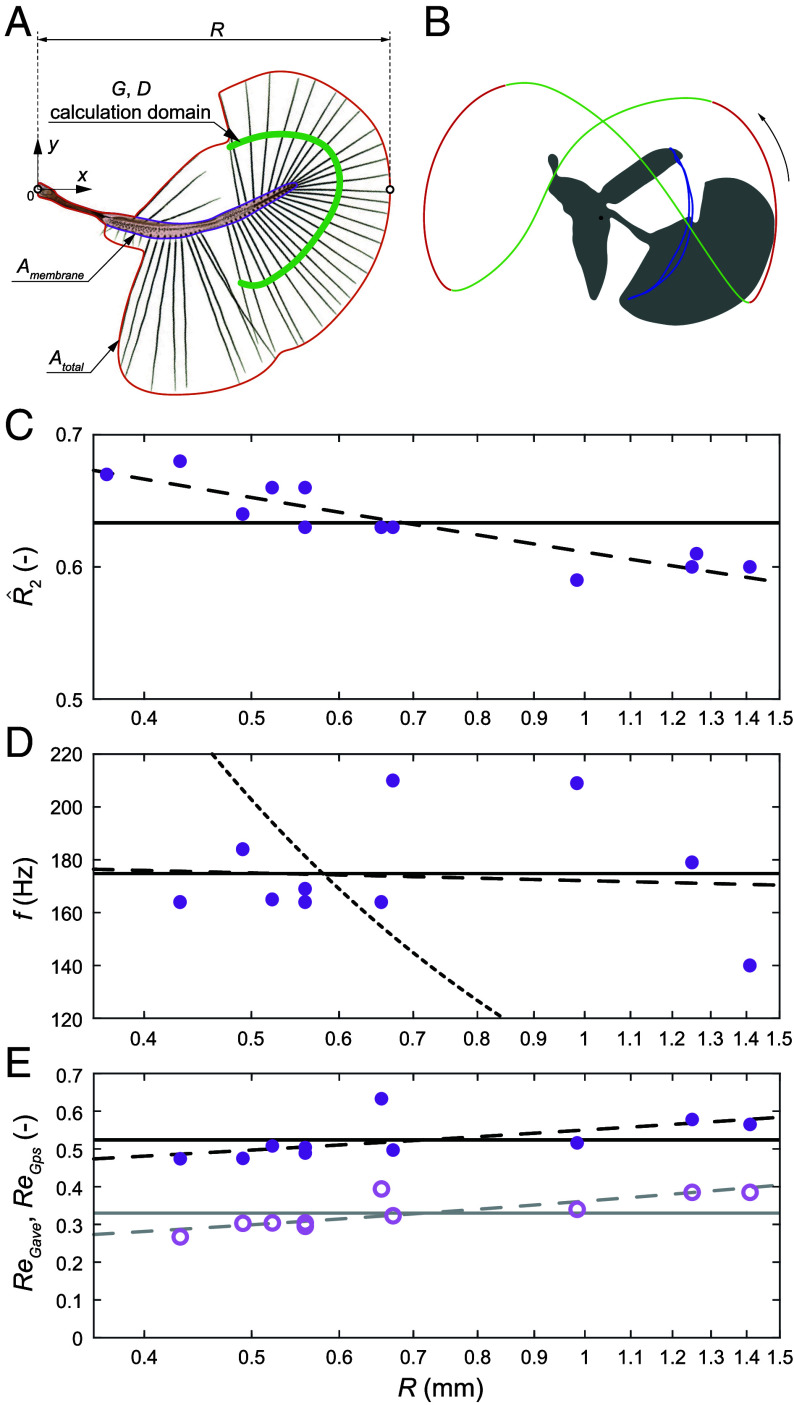
(*A*) Wing parameters: wing length R, total area $A_{\mathrm{total}}$, area without setae $A_{\mathrm{membrane}}$. (*B*) A typical wing tip trajectory in Ptiliidae consisting of two power strokes (green lines) and two recovery strokes (red lines). (*C*) Dimensionless radius of the second moment of wing area ${\hat{R}}_{2}$ as a function of wing length R. Circles show average values for each species. The dashed line is the best-fit allometric regression (slope: −0.094; *P*-value: $1.4\times 10^{-5}$ R-squared: 0.86). The horizontal solid line corresponds to the arithmetic mean value of the data points. (*D*) Wing flapping frequency f as a function of wing length R. Circles show average values for each species. The dashed line is the best-fit allometric regression (slope: −0.024; *P*-value: 0.84; R-squared: 0.0055). The black dotted line is the allometric law $f\propto R^{-1}$ (10). The horizontal solid line corresponds to the arithmetic mean value of the data points. (*E*) Gap-based Reynolds number as a function of wing length R. Pale empty circles correspond to the estimate $Re_{\mathrm{Gave}}$ based on the cycle-average flapping velocity. Bright solid circles correspond to the estimate $Re_{\mathrm{Gps}}$ at power strokes. Dashed lines are regressions for $Re_{\mathrm{Gave}}$, slope: 0.27; *P*-value: 0.004; R-squared: 0.67 and for $Re_{\mathrm{Gps}}$, slope: 0.15; *P*-value: 0.063; R-squared: 0.37. Horizontal solid lines correspond to the arithmetic mean values.

[1] $$\begin{array}{c} R_{2}={\left(\frac{\int \int_{\Omega_{w}}x^{2}\text{d}x\text{d}y}{\int \int_{\Omega_{w}}\text{d}x\text{d}y}\right)}^{1/2}, \end{array}$$

where the integration domain $\Omega_{w}$ consists of all points interior to the enveloping contour, which is visualized in Fig. 2*A* by the solid red line.

Our measurements show a variation of the normalized ${\hat{R}}_{2}=R_{2}/R$ in Ptiliidae between 0.59 and 0.68, i.e., within 15% of the mean value (Fig. 2*C*). A statistically significant power-law trend ${\hat{R}}_{2}\propto R^{-0.094}$ can explain 86% of the variance in the data, and it even extrapolates well to larger insects for which ${\hat{R}}_{2}$ is known to approach 0.5 (9). On the other hand, the absolute value of the exponent is small enough to approximate ${\hat{R}}_{2}$ as a constant parameter within the bristled-wing size domain. We will use the mean value ${\hat{R}}_{2}=0.63$.

### Wing Flapping Frequency.

The wing flapping frequency varied between f=140 and 210 Hz across all species in our data. However, it shows no obvious trend when plotted as a function of the wing length R ranging between 0.43 and 1.41 mm (Fig. 2*D*). A power-law fit suggests that $f\propto R^{\alpha }$ with the exponent α=−0.0058, which is statistically not significantly different from zero (P=0.91). It also does not align with the generally accepted interspecific allometric laws that grossly follow α≈−1 (10, 11, also see the related discussion in *SI Appendix*, section 6). The lack of a clear trend in Ptiliidae can be explained as follows. As R decreases, the fluid-dynamic loads switch from the inertial scaling $∼\rho {\left(fR\right)}^{2}$ to the viscous scaling μf, where ρ and μ are the fluid density and dynamic viscosity, respectively. This consideration was put forward (12) to explain why swimming microorganisms and large aquatic animals show two distinct allometric scalings of frequency and velocity. It is equally relevant to flying animals, but the smallest insects are situated in the transitional regime between the viscous and the inertial regimes, where neither of those power-law scalings can be used. In the absence of a clear trend, we will treat f in our analysis as a constant parameter, independent of R and equal to 174.8 Hz, which corresponds to the mean value for all points in Fig. 2*D*.

### Gap-Based Reynolds Number.

Bristled wings act as impermeable paddles for as long as flow through the gaps between bristles is effectively blocked by viscosity. This occurs when the gap-based Reynolds number, $Re_{G}=UG/\nu$, is sufficiently small, as demonstrated by Lee at al. (5) for a two-dimensional array of cylinders with diameters D<G moving at a constant velocity U in a fluid with the kinematic viscosity ν. Although $Re_{G}$ is certainly not the only parameter that controls leakiness of three-dimensional lattices in general, it is likely that this is the dominant parameter for insect wings. Let us therefore consider its allometric scaling. Let G be the average gap between bristles discussed earlier. The characteristic velocity scale U can be simply defined as the flapping cycle-average velocity at $R_{2}$. This will result in $Re_{\mathrm{Gave}}=2\pi fR_{2}G/\nu$, if the flapping amplitude is close to $180^{{}^{\circ}}$. The values thus calculated are represented by empty circles in Fig. 2*E*. They vary within ±19% of the mean value 0.33, and the allometric fit shows a weak but statistically significant trend for $Re_{\mathrm{Gave}}$ to increase with R. Variance in the gap-based Reynolds number becomes smaller if we refine the estimate using a better adapted measure for velocity scale. The wing kinematic cycle in Ptiliidae (Fig. 2*B*) consists of power strokes generating the useful aerodynamic force and recovery strokes that produce little aerodynamic force but are needed to return the wing to the starting position for the next power stroke. Therefore, low leakiness of the bristled wing only needs to be ensured during power strokes. These considerations motivate a refined estimate $Re_{\mathrm{Gps}}=2\pi \left(f/\tau_{\mathrm{ps}}\right)R_{2}G/\nu$, where $\tau_{\mathrm{ps}}$ is the time fraction of the power strokes in the full flapping cycle. The values of $Re_{\mathrm{Gps}}$ are shown in Fig. 2*E* as filled circles; they fall within ±16% of the mean value 0.52 and show weak correlation (P=0.0635). This definition also leads to a slightly smaller exponent in the allometric trend. This invariance of $Re_{\mathrm{Gps}}$ is remarkable considering that the two variable scale parameters, G and U, vary twofold across the full range of R: G varies between 7 and 14 μm, U varies between 1 and 2 ms^−1^. The nearly constant trend $Re_{G}\propto UG$ in our data agrees with the theoretical argument of Lee at al. (5). The rest of our analysis will be based on the assumption of constant $Re_{\mathrm{Gps}}=0.52$. It can be then deduced that, on average, $\tau_{\mathrm{ps}}\approx Re_{\mathrm{Gave}}/Re_{\mathrm{Gps}}=0.63$.

### Optimal Wings.

**Fig. 3.**
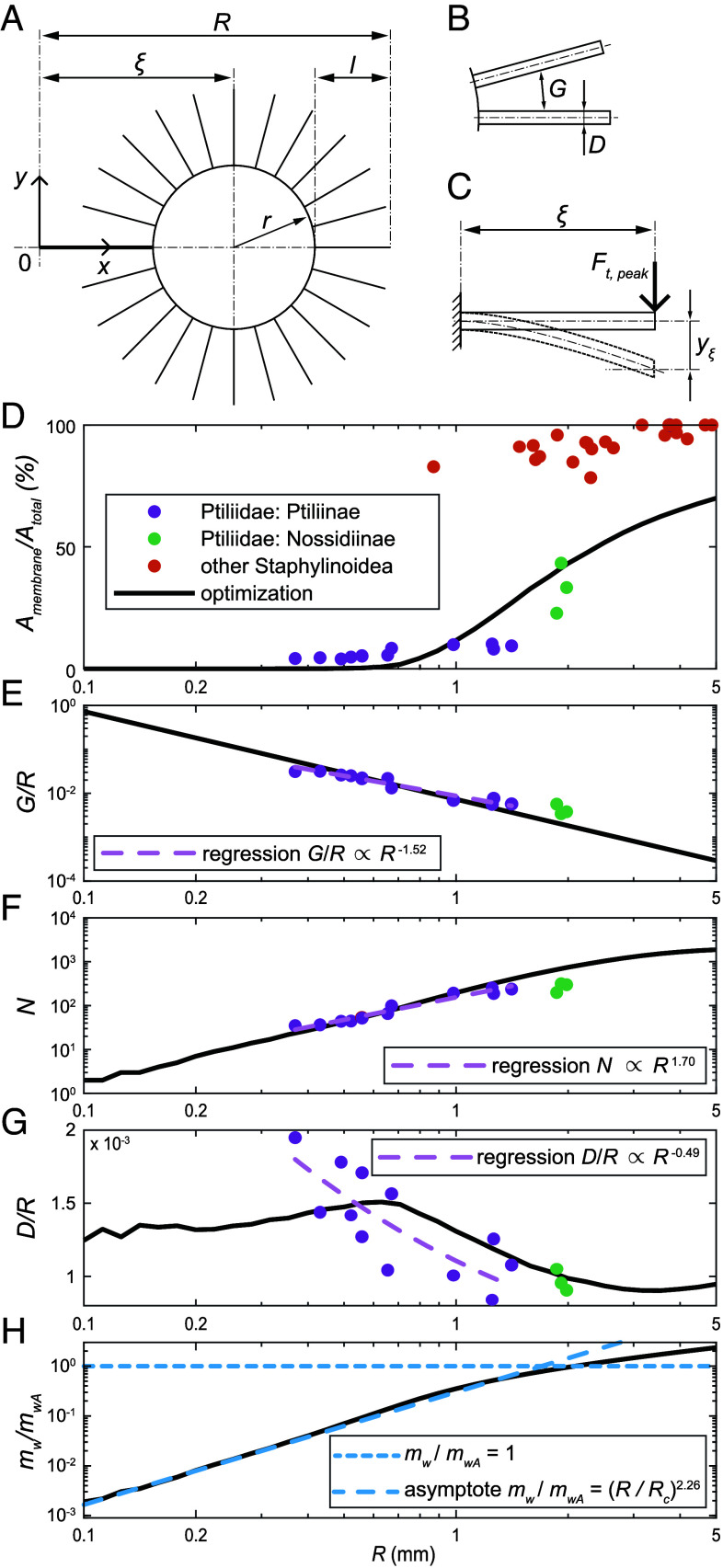
(*A*) Schematic drawing of the simplified bristled wing model. Point O is the wing shoulder joint. (*B*) Definition of the interbristle gap and bristle diameter in the simplified model. (*C*) Cantilevered beam approximation. (*D*) Optimization results in comparison with measured data for the relative membranous surface area $A_{\mathrm{membrane}}/A_{\mathrm{total}}$; (*E*) relative gap G/R; (*F*) number of bristles N, and (*G*) relative diameter D/R. (*H*) Mass of the optimal bristled wing $m_{w}$ divided by the allometric extrapolation $m_{\mathrm{wA}}=1.33{\left(A/1\;\text{mm}\right)}^{1.45}\times 1\;\mu \text{g}$ based on data for membranous-winged Staphylinoidea (4). Solid circles are data from morphological measurements; pale purple dashed lines are regression plots for Ptiliidae: Ptiliinae, solid lines are the results of optimization. Densely dashed and loosely dashed lines in (*H*) are asymptotic trends for large and small R, respectively.

Ptyloptery allows reducing the inertial loads on the flight apparatus without compromising the aerodynamic performance (4). It follows that the wing moment of inertia about the flapping axis is to be minimized, under aerodynamic and structural constraints. We consider a simplified morphological model as shown in Fig. 3 *A* and *B* and further explained in *Materials and Methods*. To specify the constraints, we postulate that i) bristled wings should generate aerodynamic forces as large in magnitude as those exerted on membranous wings of the same size for hovering, and ii) that the bristles and the central blade must be sufficiently stiff to prevent reconfiguration and concomitant loss of aerodynamic force production capacity. The first requirement is met if $Re_{\mathrm{Gps}}=0.52$ is held constant, leading to an inverse proportionality between the bristle gap size G and the wing flapping velocity $U_{\mathrm{ps}}=2\pi \left(f/\tau_{\mathrm{ps}}\right){\hat{R}}_{2}R$,

[2] $$\begin{array}{c} G=0.52\nu /U_{\mathrm{ps}}. \end{array}$$

The structural stiffness constraints lead to an algebraic relation for the geometrical parameters of the bristles (length l and diameter D) and a similar relation for the geometrical parameters of the blade (length ξ+r, width r, and thickness $h_{m}$). It follows from these constraints taken together that only two geometrical parameters are independent, and we treat R and l as the independent variables. For every value of the wing length R, we sweep over l in the range from 0 to R−ξ and find the value that minimizes the moment of inertia. The computer program is provided as part of *SI Appendix*, section 5).

The geometrical parameters were thus optimized for wings of different lengths in the range between R=0.1 mm and 5 mm. The plots in Fig. 3*D*–*G* show the variation with R of the membranous blade relative area $A_{\mathrm{membrane}}/A_{\mathrm{total}}$, the relative gap between bristles G/R, the number of bristles N, and the bristle relative diameter D/R, respectively. Continuous lines correspond to the solution for the optimization problem; point markers show data measured in beetles.

In the measured data, the relative membrane area is no greater than 10% in Ptiliinae (one of the two subfamilies of Ptiliidae), falls between 20 and 50% in Nossidiinae (the other subfamily of Ptiliidae), and is above 70% in other Staphylinoidea except Ptiliidae. The smallest wing length in the other Staphylinoidea included in our analysis is only R=0.87 mm (an unidentified species of the family Staphylinidae); the largest Ptiliinae wing is as long as R=1.41 mm (*Acrotrichis intermedia*), while Nossidiinae wings are situated at R≈2 mm. Thus, essentially membraneous and bristled wings coexist in the range of R between 0.87 and 2 mm. The optimization model consistently suggests that the shortest wings must have the least relative membrane area; R=1 mm-long wings have $A_{\mathrm{membrane}}/A_{\mathrm{total}}=12\%$. For larger wings, optimization suggests a gradual increase of $A_{\mathrm{membrane}}/A_{\mathrm{total}}$ passing through 40% at R=2 mm (Nossidiinae size) up to 70% at R=5 mm (larger Staphylinidae size). Our analytical model does not include veins stiffening the membrane, which explains the delayed transition to fully membranous wings. The reasons why optimization produces this behavior of $A_{\mathrm{membrane}}/A_{\mathrm{total}}\left(R\right)$ can be understood from the tendencies of G/R, N, D/R, and $m_{w}$ discussed next.

Let us first focus on the parameters that characterize the strongly bristled wings of Ptiliidae. Optimal scaling of the average gap between bristles follows directly from the aerodynamic condition [**2**]: smaller wings have proportionally wider spacing of bristles, which means that the dimensionless ratio varies as $G/R\propto R^{-2}$ under the assumptions made. An allometric regression through the measurement data points (in Fig. 3*E*) shows a similar trend with a somewhat smaller slope of −1.52 on average (lower bound −1.83; upper bound −1.28; $P=1.78\times 10^{-7}$).

The number of bristles N scales as the inverse of G/R, from geometrical considerations, yielding $N\propto R^{2}$ (Fig. 3*F*). In Ptiliinae, N varies between 35 in the smallest wings of *S. musawasensis* to 238 in *A. intermedia*. In Nossidiinae, N can be as large as 300. The analytical model results in an increasing trend N(R) that agrees with the data remarkably well for the small R, where $A_{\mathrm{membrane}}/A_{\mathrm{total}}$ is also well characterized. An allometric trend through the measured data has the slope estimate of 1.71, which is even closer to the theoretical quadratic scaling (lower bound 1.48; upper bound 2.00; $P=3.85\times 10^{-8}$).

The optimal relative diameter of the bristle, D/R, is influenced by all factors included in the model. However, this quantity shows surprisingly little variation. We display it on a semilog scale in Fig. 3*G*. All measured values fall between 0.0005 and 0.002. There is, nevertheless, a statistically significant allometric trend, with a slope of −0.49 (lower bound −0.84; upper bound −0.22; P=0.004). In fact, the analytical model reveals a crossover of two tendencies. For wings shorter than 0.7 mm, the membrane is fully reduced and the bristle length becomes simply proportional to the wing length. All aerodynamic load is carried by the bristles. For wings longer than 0.7 mm, bristles have a progressively smaller load-carrying capacity as R increases. Despite this complex trend, given the small range of variation of D/R, we conjecture that bristles must always be of the order one-tenth percent thick, relative to the wing length, in all species.

Minimization of the wing inertia is achieved mainly through decreasing of the wing mass by reducing the membrane. Moments of inertia are difficult to measure; at the same time, data are readily available on the wing mass in membranous-winged species (4). It is insightful to compare the mass of optimized wings, $m_{w}$, with a trend for larger beetles, $m_{\mathrm{wA}}=1.33{\left(A/1\;\text{mm}\right)}^{1.45}\times 1\;\mu \text{g}$, where A is the wing area (which we estimate here as $\pi {\left(R-\xi \right)}^{2}$). This is presented in Fig. 3*H* as the ratio $m_{w}/m_{\mathrm{wA}}$ vs. R. It is of order unity on the large-size end of the plot, and falls by three orders of magnitude as R decreases, which means that ptyloptery does reduce the wing mass substantially. The plot also highlights the presence of two different asymptotic scalings for two different conceptual designs: the empirical allometry for membranous wings is $m_{w}\left(R\right)∼m_{\mathrm{wA}}\left(A\left(R\right)\right)$; for wings that consist mainly of bristles, the scaling at small R approaches a power law $m_{w}\left(R\right)∼{\left(R/R_{c}\right)}^{2.26}\times m_{\mathrm{wA}}\left(A\left(R\right)\right)$ with $R_{c}=$1.7 mm. Both types of design ensure the necessary aerodynamic capability and structural strength. But, because of the factor $R^{2.26}$, membranous wings are lighter at larger sizes, and bristled wings are lighter at smaller sizes. The asymptotic scalings intersect at $R=R_{c}$, and near this size the optimum is achieved through a combination of membranous and bristled designs, i.e., with $A_{\mathrm{membrane}}/A_{\mathrm{total}}$ not close to 0 or 100%. The exact location of the crossover point $R_{c}$ must depend on specific morphological and physiological traits; therefore, it may vary across different taxonomic groups.

The alignment between the optimization results and the observed allometric trends supports the key hypotheses of optimization: minimization of wing inertia under the aerodynamic and structural stiffness constraints. The spacing of the bristles is limited by the maximum aerodynamically permissible gap between them, while their diameter is selected to provide adequate bending stiffness. This design prevents bending reconfiguration and the concomitant loss of aerodynamic force. However, the wings of larger insects require a disproportionately greater number of bristles ($N\propto R^{\left[1.48,2\right]}$), which can only fit if the perimeter of the central membranous blade is sufficiently long. This explains why the largest practical bristled wings in Ptiliidae are R=2 mm long, beyond which bristles become too short and tightly packed so that they cannot help reduce inertia. The opposing limit suggested by the model is that the wings that are only 0.1 mm long can have only two bristles.

Wing shapes in other insects that have bristled wings, such as thrips and wasps, are expected to conform to the same underlying principles. In *SI Appendix*, section 6, we present optimal trends for Mymaridae (fairy wasps) derived using the same technique. Our analysis reveals differences between Mymaridae and Ptiliidae in certain scaling laws, which may be accounted for by hypothesizing a distinct allometric scaling relationship for wing flapping frequency. This hypothesis is indirectly supported by observed allometric trends of the number of bristles and spacing between them. However, due to the absence of kinematic data, direct testing of this hypothesis remains a subject for future research.

## Materials and Methods

This section begins with a description of the morphological and kinematic measurement techniques employed. Then it provides details on the mathematical models used within the optimization.

### Specimens.

We examined morphological and kinematic parameters in 38 species of Staphylinoidea (families Ptiliidae, Staphylinidae, Hydraenidae, Leiodidae, Jacobsoniidae; see Datasets S1 and S2). The full list is contained in Datasets S1 and S2. We obtained most of our data from 23 species collected in Moscow Oblast (Russia) and Cát Tiên National Park (Vietnam). We also added published data (8, 13–18).

### Morphometrics.

Selected specimens were fixed in 70% ethanol for morphometrics. Body lengths were measured on micrographs obtained using a scanning electron microscope (SEM) JSM-6380 (JEOL) for the smallest species and photos obtained by light microscopy and camera (Nikon 1 V3) with macrolens (Nikon 1 NIKKOR 60 mm) for the larger species.

For wing dissection, fixed insects were softened for 24 h in a 5:5:1 mixture of ethanol, glycerol, and water. The wings were dissected and washed in 100% ethanol twice for 24 h. The wings of ptiliids and other smallest staphylinoids were infiltrated with Eukitt UV (Orsatec) resin for 24 h and mounted on permanent slides for light microscopy in the same medium. Preparations of the large wings were made in Euparal resin. During preparation to the SEM, dissected and washed wings were immersed in a droplet of distilled water on a glass slide glued to the microscope stage with varnish and then dried at the critical point with a Hitachi HCP-2 dryer. The mounted samples were sputter-coated using a Giko IB-3 ion-coater. Micrographs were obtained using SEM JEOL JSM-6380 and FEI Quattro S. Using wing photos obtained by light microscopy, we measured distances and areas that can be inferred from planar views of the entire wing, as detained in Fig. 2*A*. Using SEM micrographs, we measured the dimensions of individual setae on the sample size of at least 10 in each species.

Using those measurements, the following characteristics were calculated. Part of wing area occupied by setae is the ratio of $A_{\mathrm{total}}-A_{\mathrm{membrane}}$ over $A_{\mathrm{total}}$, in per cent. The mean chord length is defined as $c=A_{\mathrm{total}}/R$. The bristle diameter D in this work refers to the average stem diameter of the setae (bristles) at the middle of their length. The average gap between setae is calculated as $G=\sigma -D_{\mathrm{eff}}$, where setae density σ is the average distance between the setal axes at the middle of their length, and $D_{\mathrm{eff}}=D_{\mathrm{stem}}+k_{\mathrm{eff}}\left(D_{\mathrm{outgr}}-D_{\mathrm{stem}}\right)$ is the aerodynamically effective bristle diameter. The latter formula uses measurements of stem diameter $D_{\mathrm{stem}}$ and secondary outgrowth diameters $D_{\mathrm{stem}}$ for setae in each of the species, and scales them using a constant coefficient $k_{\mathrm{eff}}=0.418$, which is based in on earlier measurements in *P. placentis* (19).

To estimate gap Reynolds number $Re_{G}$, we choose wing velocity at the radius of the second moment of wing area during power stroke $U_{\mathrm{ps}}$ as the characteristic velocity, yielding $Re_{\mathrm{Gps}}=U_{\mathrm{ps}}G/\nu$.

### Kinematic Reconstruction.

Free flight videos of insects were recorded using a setup of synchronized high-speed cameras Evercam 4000 at 3,000 to 5,200 FPS in small glass chambers in transmitted infrared light. The setup scheme was the same as described earlier (20), with the exception of the use of macrolenses. We measured wingbeat frequency f in each recorded species and relative duration of power stroke $\tau_{\mathrm{ps}}$ in ptiliids. In seven Ptiliidae species, which were recorded on two cameras, we reconstructed in 3D wingtip trajectories during at least 3 and up to 10 cycles and calculated the velocities at $R_{2}$.

### Statistical Analysis.

The average values of morphometric and kinematic characteristics for each species were calculated as medians due to the moderate sample size. The median values were analyzed by standardized major axis estimation among groups which was performed in the R environment using SMATR package (21).

### Morphological Model.

The wing length R is treated as a fixed parameter and a proxy for the animal size during the optimization process. Geometrical similarity of wing and body shapes is assumed. In particular, c∝R and $L_{b}\propto R,$ where $L_{b}$ is the body length (*SI Appendix*, section 2).

We consider a simplified bristled wing model that consists of a circular flat membraneous central part and a fringe of bristles; see Fig. 3*A*. The bristles are modeled as circular cylinders, all identical and having the sectional diameter D. We deliberately neglect such features of the real bristles as the conical shape and secondary outgrowths. The planar shape is described by the following parameters, which are allowed to vary during the optimization process: radius of the central blade r; thickness of the membraneous central part $h_{m}$; bristle length l; number of bristles N; distance from the wing hinge point to the center of the membraneous part ξ. The wing length is therefore equal to R=ξ+r+l. Let $\hat{\cdot }$ denote distances nondimensionalized by R. We then obtain $\hat{\xi }+\hat{r}+\hat{l}=1$. The parameter $\hat{\xi }$ can be evaluated through the radius of the second moment of wing area as $\hat{\xi }=0.2+0.4\sqrt{5{\hat{R}}_{2}^{2}-1}$, equaling 0.597 in our calculation.

### Inertial Properties.

The moment of inertia about the Oy axis (Fig. 3*A*) is calculated as a sum of contributions from the central membranous part and the bristles,

[3] $$\begin{array}{c} J_{w}=J_{m}+J_{b}. \end{array}$$

The membranous part is a disk with constant thickness $h_{m}$, yielding

[4] $$\begin{array}{c} J_{m}=m_{m}\left(\xi^{2}+r^{2}/4\right), \end{array}$$

where $m_{m}=\rho_{w}h_{m}\pi r^{2}$ is the mass of the membranous part. The bristles are represented by thin rods, which are uniformly distributed along the periphery. Thus, their contributions sum up as

[5] $$\begin{array}{c} J_{b}=\sum_{j=1}^{N}m_{b}\left(\frac{1}{12}l^{2}\cos^{2}\beta_{j}+x_{j}^{2}\right), \end{array}$$

where $m_{b}=\rho_{w}l\pi D^{2}/4$ is the total mass of one bristle, $\beta_{j}=2\pi \left(j-1\right)/N$ is the bristle orientation angle and $x_{j}=\xi +\left(r+l/2\right)\cos\beta_{j}$ is its center-of-mass coordinate. The wing mass, similarly, equals $m_{w}=m_{m}+Nm_{b}$.

### Kinematical Model.

The aerodynamic forces are maximal during the power stroke. Therefore, the characteristic velocity in the optimization problem is

[6] $$\begin{array}{c} U_{\mathrm{ps}}=2\Phi \left(f/\tau_{\mathrm{ps}}\right){\hat{R}}_{2}R. \end{array}$$

On the basis of our measurements in Ptiliidae species, throughout the optimization process we set constant wing flapping amplitude limited by clapping Φ=π, frequency f=174.8 Hz, relative duration of power strokes $\tau_{\mathrm{ps}}=0.63$ and relative radius of the second moment of wing area of the wing ${\hat{R}}_{2}=0.63$.

### Aerodynamic Considerations.

We limit the gap-based Reynolds number $Re_{\mathrm{Gps}}$ to a value that ensures that bristled wings generate aerodynamic forces close in magnitude to those produced by similarly sized membranous wings. This condition allows us to use general trends of aerodynamic forces across wings of various sizes, primarily based on data from membranous wings.

We selected eleven species for which published CFD data are available for time-varying aerodynamic forces during hovering (2, 4, 22–27). For each, we sampled peak aerodynamic force magnitude $F_{\mathrm{peak}}$ per wing and the respective velocity $U_{\mathrm{ps}}$. From morphological data of the same species, we determined the wing area A, wing length R mean aerodynamic chord length c=A/R and, using the velocity $U_{\mathrm{ps}}$, the representative Reynolds number

[7] $$\begin{array}{c} Re_{\mathrm{ps}}=U_{\mathrm{ps}}c/\nu , \end{array}$$

and the peak aerodynamic force coefficient

[8] $$\begin{array}{c} C_{\mathrm{Fpeak}}=\frac{2F_{\mathrm{peak}}}{\rho U_{\mathrm{ps}}^{2}A}, \end{array}$$

where ρ is the density of air. Next, we approximated the pairs $\left(Re_{\mathrm{ps}},C_{\mathrm{Fpeak}}\right)$ with polynomial function in the form of a Laurent series containing two terms,

[9] $$\begin{array}{c} C_{\mathrm{Fpeak}}=\frac{C_{-1}}{Re_{\mathrm{ps}}}+C_{0}. \end{array}$$

The coefficients are fitting parameters, and from the $L^{1}$ residual error norm minimization, we found $C_{-1}=15.20$ and $C_{0}=1.63$. More detailed information about the data and the fitting procedure can be found in *SI Appendix*, section 1. Finally, in the optimization loop, Eqs. **7** and **9** were used with input data characterizing the simplified model: $A=\pi r^{2}$ and $U_{\mathrm{ps}}$ as defined by Eq. **6**.

### Structural Stiffness Considerations.

We neglect torsion and require that the wing does not bend excessively. A simple cantilever model (Fig. 3*C*) is sufficient for an order-of-magnitude estimation of the wing deflection at the central point, $y_{\xi }=F_{w}\xi^{3}/3EI_{m}$, where the aerodynamic loading is reduced to a point force $F_{w}$ applied at the shoulder distance ξ. The material Young’s modulus is denoted as E. The sectional second moment of area $I_{m}=K_{m}c_{m}h_{m}^{3}$ scales in proportion with the membraneous part mean chord length $c_{m}$ and the cube of the wing effective thickness $h_{m}$, with a constant proportionality coefficient $K_{m}$ that we set equal to that of a constant rectangular cross section, $K_{m}=1/12$. It is customary to define the mean chord length as the area divided by the length of the wing. Here, we use a similar definition, but based on the central blade parameters, $c_{m}=\pi r^{2}/\left(\xi +r\right)$. We require that all optimized wings bend by a constant fraction of the wing length, $y_{\xi }=\hat{\gamma }R$, where $\hat{\gamma }=\;\arctan\left(10^{{}^{\circ}}\right)$. The equations are resolved with respect to $h_{m}/R$.

The bristles should be sufficiently stiff to sustain aerodynamic loading. If they bend, the gap between them may increase. We constrain the maximum allowed linear deflection as a constant fraction of the gap, $y_{b}=2\eta G$, with η=0.18, which is based on *P. placentis* measurements (*SI Appendix*, section 4). The deflection of the end of a cantilevered bristle under a uniformly distributed load q is estimated using the linear beam theory as $y_{b}=ql^{4}/8EI_{b}$, where the sectional second moment of area is equal to $I_{b}=\pi a^{4}/4$. The aerodynamic force per unit length of the bristle is estimated as

[10] $$\begin{array}{c} q=\frac{F_{w}}{\mathrm{Nl}}\left(1-\frac{r^{2}}{{\left(r+l\right)}^{2}}\right). \end{array}$$

## Supplementary Material

Appendix 01 (PDF)

Dataset S01 (XLSX)

Dataset S02 (XLSX)

## Data Availability

All study data are included in the article and/or supporting information.
