# Supplementary material for: Scaling and mechanical optimality of bristled wings in microinsects
Source: Proc Natl Acad Sci U S A. 2025 Aug 22;122(34):e2506403122. doi: 10.1073/pnas.2506403122 (PMC12403095; doi:10.1073/pnas.2506403122)
Supplement: Supplementary file 1 — Appendix 01 (PDF) [file pnas.2506403122.sapp.pdf]

Supporting Information for

Scaling and mechanical optimality of bristled wings in microinsects

Dmitry Kolomenskiy, Sergey E. Farisenkov, Pyotr N. Petrov and Alexey A. Polilov

Corresponding Author: Dmitry Kolomenskiy.
E-mail: d.kolomenskiy@skoltech.ru

This PDF file includes:

Supporting text
Figs. S1 to S6
Table S1
Legends for Dataset S1 to S2
SI References

Other supporting materials for this manuscript include the following:

Datasets S1 to S2

Supporting Information Text

1. Estimation of the aerodynamic force

Time profiles of the aerodynamic force during hovering typically have two distinct peaks, one on downstroke and the other on upstroke. They are about twice as large in magnitude as the time-average force. The precise value depends, among other parameters, on the wing velocity, i.e., estimation of the peak aerodynamic force requires knowledge of the wing kinematics. We construct an empirical formula using the kinematic measurements and CFD calculations of the force using data from literature (1–8), combining bristled and membranous wing data. Under the condition that the gap Reynolds number Re_G is sufficiently small to block the flow between bristles, the two kinds of wings produce aerodynamic forces of the same order of magnitude, that scale similarly with the body size. Moreover, the data sources encompass a variety of species, not only coleopterans. Thus we obtain an amalgamated dependence of the aerodynamic force coefficient as a function of the Reynolds number Re_{peak} of a wing at maximum speed of translation. It can be regarded as an aerodynamic property of the wing of a selected representative baseline shape at the baseline angle of attack. The dispersal of the individual data points relative to the trends will reflect the deviation of the individual wing shapes and kinematic conditions from the baseline, as well as the effect of ptiloptery.

Supplementary Table S1 summarizes the main geometrical and kinematic parameters of wings used in the peak force coefficient calculations. The dimensional force data are obtained from the sources by either directly digitizing the plots of dimensional vertical and horizontal forces of a pair of wings $F_v(t)$ and $F_h(t)$, or recalculating from dimensionless coefficients, depending of the presentation format in the source. We then calculate the force magnitude $F_t(t) = \sqrt{F_v^2 + F_h^2}$. In all cases it has two peaks, $F_{t,peak,1}$ and $F_{t,peak,2}$, achieved in the middle of downstroke and upstroke translational phases. The translational velocity is evaluated as $U(t) = \hat{R}_2 R \sqrt{\dot{\phi}^2 + \dot{\theta}^2}$, where dots denote time derivatives, obtained by numerical differentiation of wing positional $\phi(t)$ and elevation $\theta(t)$ angles acquired from the same sources. We then determine the velocity magnitude values U_1 and U_2 at the two time instants corresponding to the peaks of aerodynamic force. These are used in the calculation of the Reynolds number at the geometrical radius of the 2nd moment of wing area,

$$Re_{2,peak,j} = \frac{U_j c}{\nu}, \quad j = 1, 2. \qquad [1]$$

The force coefficient of a wing is then determined,

$$C_{F,peak,j} = \frac{F_{t,peak,j}/2}{0.5 \rho U_j^2 A_w}, \quad j = 1, 2, \qquad [2]$$

where $A_w = cR$ is the wing area. The division by 2 accounts for $F_{t,peak,j}$ being the force generated by a pair of wings. The data pairs $(Re_{2,peak,j}, C_{F,peak,j})$ are displayed in Supplementary Figure S1 using asterisk markers.

We fit a trend in the form of Oseen approximation

$$C_{F,peak} = \frac{C_{-1,peak}}{Re_{2,peak}} + C_{0,peak}. \qquad [3]$$

The coefficients take the values $C_{-1,peak} = 15.20$ and $C_{0,peak} = 1.63$. These values minimize the L^1 residual error norm, as we find from a parameter sweep in a two-dimensional domain $(C_{-1,peak}, C_{0,peak}) \in [0, 100] \times [0, 10]$ with 0.01 increment. The approximation (3) is asymptotically correct in the limit of small Re and remains valid at finite Re up to the onset of turbulence, which occurs at $Re > 100$ on flapping wings (insects larger than the fruit fly). Terms with higher powers of Re can be incorporated to make the fit more accurate at higher Re. In our study, the simple two-parameter fit (3) is sufficient, because variations of the force coefficient due to turbulence are not relevant.

Table S1. Geometrical and kinematic parameters of wings used in peak force coefficient calculations: wing length R, mean chord length c, dimensionless geometrical radius of the 2nd moment of wing area $\hat{R}_2$ normalized by the wing length, and the wing beat frequency f.

Species	Source	R (mm)	c (mm)	$\hat{R}_2$ (-)	f (Hz)
Anbremia sp.	(6)	1.33	0.38	0.63	238
Bombus ignitus	(4)	15.2	4.2	0.57	136
Dasyhelea flaviventris	(6)	0.92	0.27	0.6	723
Drosophila virilis	(6, 7)	2.90	0.78	0.59	161
Encarsia formosa	(2, 6)	0.61	0.23	0.64	361
Eristalis tenax	(5, 6)	11.20	2.98	0.55	164
Forcipomia gloriose	(6)	1.30	0.29	0.6	1117
Frankliniella occidentalis	(6)	0.76	0.39	0.59	239
Liriomyza sativae	(1, 6)	1.44	0.42	0.59	260.8
Paratuposa placentis	(3)	0.493	0.24	0.63	171
Trypoxylus dichotomus	(8)	51.2	15.5	0.6	37.04

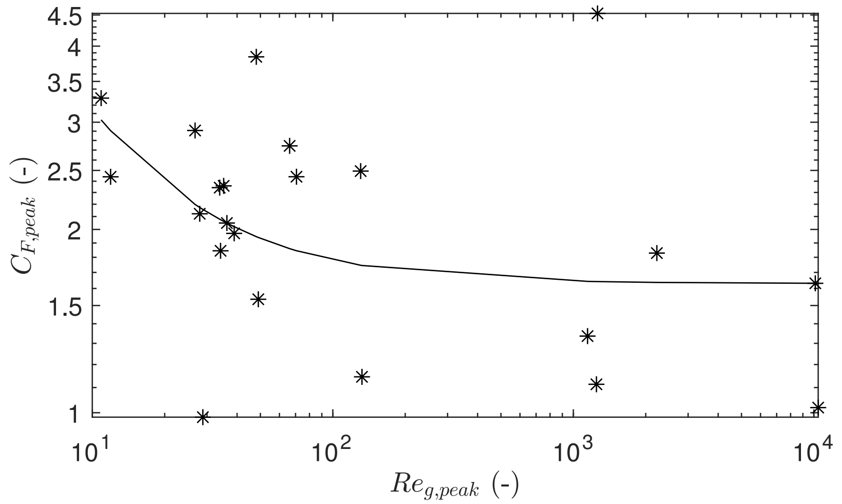

Fig. S1. Fitting of the aerodynamic force coefficient as a function of the Reynolds number.

2. Allometry of wing length

Regression analysis of the wing length as a function of body length was done for two groups: Ptiliidae and membranous-winged Staphylinoidea. For Ptiliidae, the scaling is close to isometric: the wing length R scales with the body length L_b as $R \propto L_b^\alpha$ with the exponent estimated as $\alpha = 1.16$ (lower bound 0.99; upper bound 1.37; $p = 5.35 \times 10^{-9}$). For the membranous-winged Staphylinoidea, the exponent is less close to unity but also data are more dispersed (exponent α estimate 0.73; lower bound 0.20; upper bound 1.82; $p = 0.0195$). The respective plots are shown in Supplementary Figure S2.

3. Note on the geometrical radius of the 2nd moment of wing area of the wing in relation to the aerodynamic force estimation

The approach of using the 2nd moment of wing area to define the characteristic velocity of a flapping wing was introduced by Weis-Fogh (9) and Ellington (10) in the context of insect flight aerodynamics. But it originates from earlier blade-element calculations for propellers, which offer a simple explanation why R_2, the radius of the 2nd moment of wing area, is an important parameter for the aerodynamic force estimation. A brief summary of it is provided in this section.

We define the aerodynamic force coefficient of the wing using the velocity U at radius R_2 as the reference velocity,

$$C_F = \frac{2F}{\rho U^2 A_w} \qquad [4]$$

This choice of reference velocity ensures that the numerical value of the force coefficient of the entire wing is close to the sectional aerodynamic force coefficient. This property follows from the blade element analysis. This following discussion will apply to, both, the vertical and the horizontal components of the force. Therefore, we refer to the aerodynamic force as a scalar component F, without specifying the direction.

Let us consider a rotary wing with a constant spanwise distribution of sectional aerodynamic force coefficient (see Supplementary Figure S3). Let Ω be the angular velocity of rotation. Then, the reference velocity to be used in (4) is equal to $U = \Omega R_2$. The sectional aerodynamic force F_{sec} is related to its coefficient C_{Fsec} as

$$F_{sec} = C_{Fsec} \frac{\rho \left(\Omega r\right)^2}{2} c(r). \qquad [5]$$

The aerodynamic force of the entire wing is calculated through spanwise integration,

$$F = \int_0^R C_{Fsec} \frac{\rho \left(\Omega r\right)^2}{2} c(r) \mathrm{d}r = C_{Fsec} \frac{\rho \Omega^2}{2} \int_0^R r^2 c(r) \mathrm{d}r. \qquad [6]$$

Noticing that chord length $c(r)$ is the chordwise distance between the leading edge and the trailing edge at a fixed spanwise location r, we find that $\int_0^R r^2 c(r) \mathrm{d}r = R_2^2 A_w$, where A_w is the wing area. We obtain

$$F = C_{Fsec} \frac{\rho \left(\Omega R_2\right)^2}{2} A_w. \qquad [7]$$

Substituting (7) into (4) we see that

$$C_F = C_{Fsec}. \qquad [8]$$

In a situation when the sectional force coefficient distribution is not uniform, the equality (8) becomes approximate, which means that aerodynamic force of the wing can be estimated by considering the sectional flow parameters at R_2.

4. Individual bristle deflection estimation

The bristles must be sufficiently stiff to sustain aerodynamic loads. Sufficient stiffness means that bending deformations do not significantly widen the gap between bristles. In other words, bending deflection of the bristles must be much less than the gaps between them. In our model, we introduce a parameter

$$\eta = \frac{y_b}{2G}, \qquad [9]$$

where y_b is the maximal linear deflection of the tip of a bristle which typically occurs during the translation phase of flapping, and G is the mean gap between the centers of two neighbor bristles for a wing at rest. For wings with negligibly small central membrane, $2G$ is the mean distance between the tips of two neighbor bristles, which means that $\eta = 0.5$ would correspond to two bristle tips touching each other if they bend in opposite directions. The latter is an unlikely event because, in that case, the gap doubles and every second bristle becomes unnecessary. It follows that η must be less than 0.5.

We estimated η for Ptiliidae using high-speed video data of *Paratuposa placentis*. We selected frames that show wings projected edge-on during upstroke and downstroke translation. For each frame, we calculated the angle between the membranous part and the bristled part, using extremities as base points, see Supplementary Fig. S4. Let us denote this angle as θ_{bu} during upstroke and θ_{bd} during downstroke. The value of θ_{bu} averaged over 5 measurements is equal to 2.82 ± 0.58 deg (average $\pm$ standard deviation). The value of θ_{bd} averaged over 7 measurements is equal to -0.57 ± 2.74 deg. The angular deflection amplitude is equal to $\Delta\theta_b = \theta_{bu} - \theta_{bd} = 3.39$ deg.

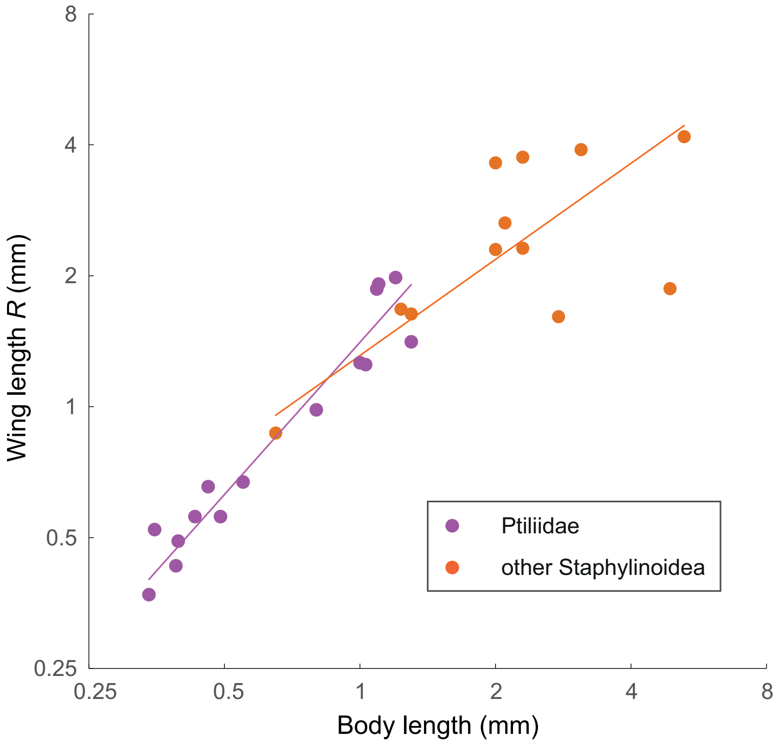

Fig. S2. Regression of the wing length R as a function of body length, for Ptiliidae and membranous-winged Staphylinoidea. Circles show measured data, lines show regression plots.

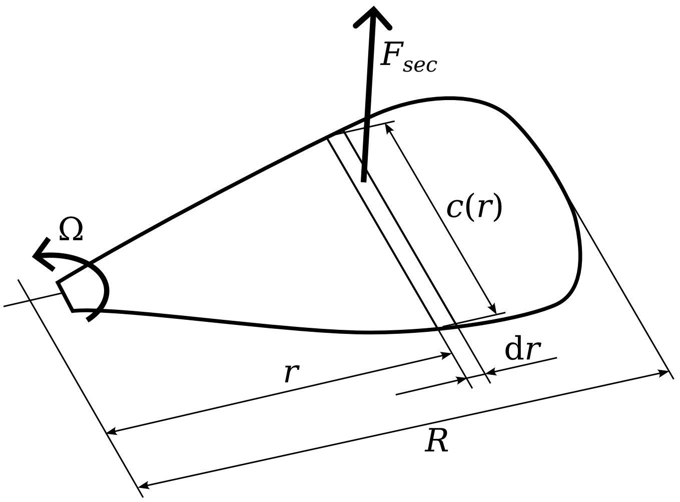

Fig. S3. Schematic drawing of a rotary wing, which serves a simplified model for the ‘translation’ phase of a flapping wing.

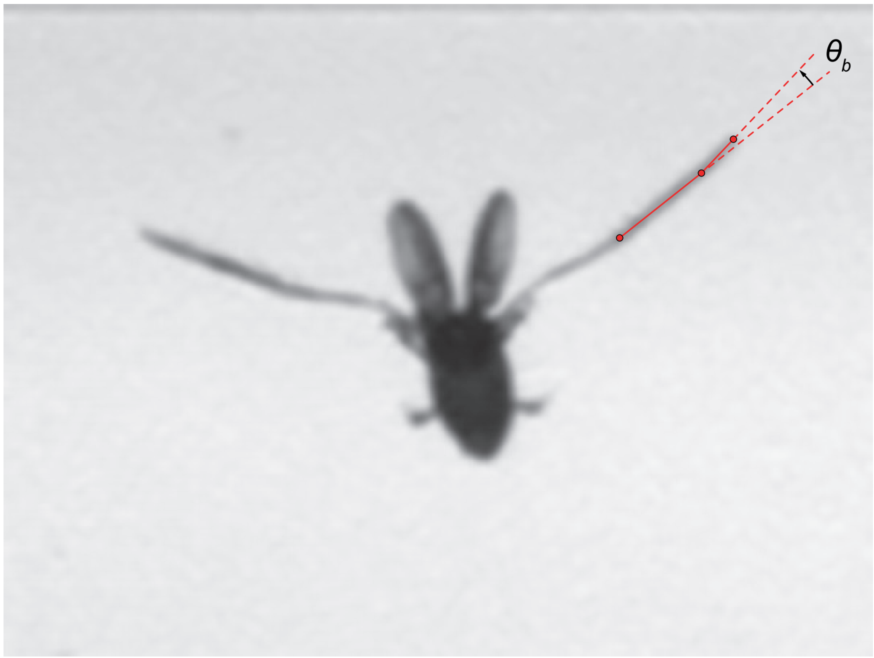

Fig. S4. Angular deflection of bristles relative to the membranous part of the wing of *P. placentis* during downstroke.

This measured angular flexion amplitude is converted into linear deflection of the bristle tip,

$$y_b = l \sin(\Delta\theta_b/2), \quad [10]$$

where l is a model bristle length evaluated using the idealized model shown in Fig. 3A of the main text as $l = \sqrt{A_{total}/\pi} - \sqrt{A_{membrane}/\pi}$, using values of $A_{total} = 0.118 \times 10^{-6}$ m^2 and $A_{membrane} = 0.04 A_{total}$ that correspond to *P. placentis*. Substituting these estimates and the average gap $G = 12.79$ μm into (9), we find

$$\eta = 0.18. \quad [11]$$

This is the value that we use for Ptiliidae calculations in the main text, in particular in Fig. 3.

Since we determined the value of η using data for only one species, it is important to consider sensitivity of the model to this parameter. Supplementary Figure S5 shows plots of $A_{membrane}/A_{total}$, G/R, N, D/R and m_w/m_{wA} (in the same order as in Fig. 3 in the main text) for three values of η: 0.5 is the aerodynamically justified upper bound as explained above, 0.18 is the empirical value for Ptiliidae and 0.05 is an arbitrary small value. The direct effect of prescribing a smaller value of η in the optimization is to reduce tolerance on bending deflection of the bristles, i.e., to demand greater stiffness which is achieved by enlarging the diameter D. This is clearly visible in Supplementary Figure S5D for the wings that are essentially bristled. The large wings that are essentially membranous are not sensitive to η, which is expected. As larger values of D/R make thicker and, consequently, heavier bristles, they become less helpful in lightening the wing (see Supplementary Figure S5E). Therefore, optimal wings with smaller η tend to have a larger membrane area for the same size R (see Supplementary Figure S5A). The relative gap G/R and the number of bristles N are not sensitive to η.

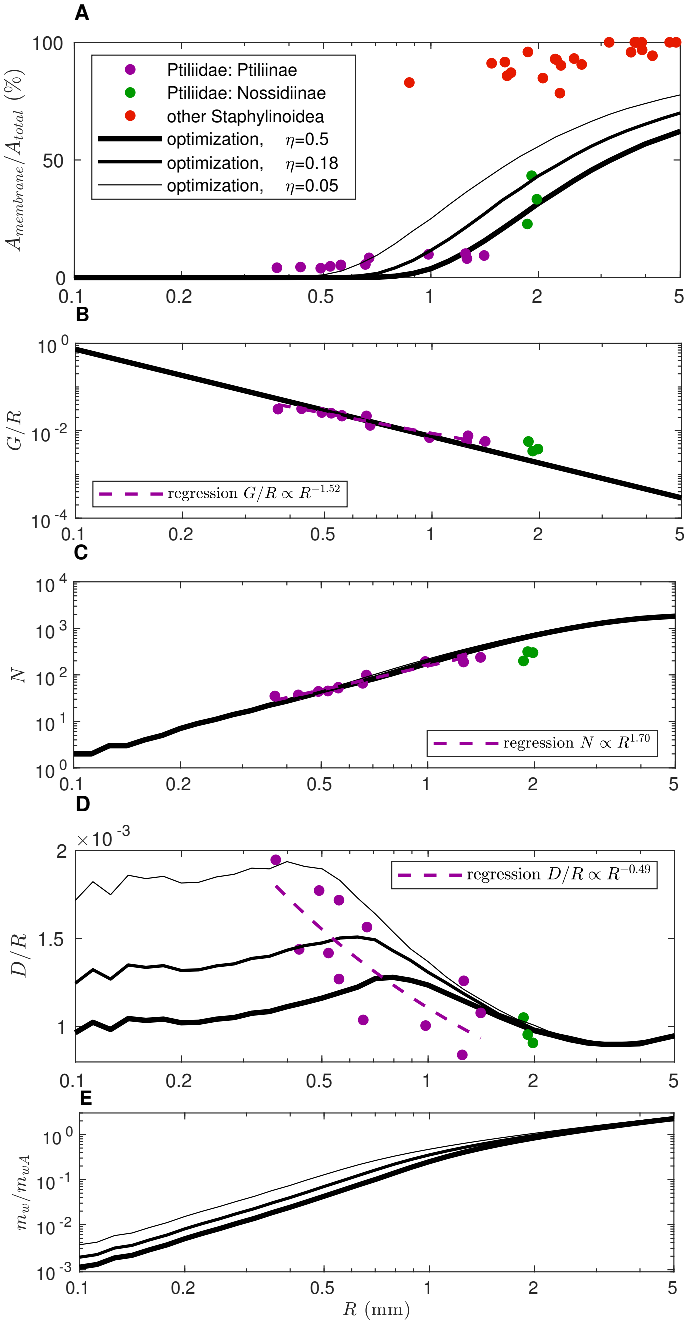

Fig. S5. The effect of bristle stiffness parameter η on the optimization results: (A) relative membranous surface area $A_{membrane}/A_{total}$; (B) relative gap G/R; (C) number of bristles N and (D) relative diameter D/R. (E) Mass of the optimal bristled wing m_w divided by the allometric extrapolation $m_A = 1.33(A/1\,\text{mm})^{1.45} \times 1\,\mu\text{g}$ based on data for membranous-winged Staphylinoidea (3). Solid circles are data from morphological measurements; pale dashed lines are regression plots for Ptiliidae: Ptiliinae, solid lines are the results of optimization using $\eta = 0.5, 0.18$ (as measured in *P. placentis*) and 0.05.

5. Bristled-wing optimization program listings

In this section, we list the program used for the line plots in Fig. 3 in the main text of the article. The optimization procedure was implemented in Matlab. It consists of the main program and a function that calculates the moment of inertia.

```
% script_opt_design.m : Optimization of bristled wings
clearvars;
close all;

% Vector of wing length (m)
Rvec = 10.^(-4:0.05:-2);

% Loop for all values of the wing length
for j = 1:length(Rvec)

    % Constant parameters
    % Wing length (m)
    R = Rvec(j);
    % Flapping frequency (Hz)
    f = 174.8;
    % Flapping amplitude (rad)
    Phi = pi;
    % Air density (kg/m^3)
    % see https://doi.org/10.1038/s41586-021-04303-7
    rho = 1.197;
    % Air kinematic viscosity (m^2/s)
    % see https://doi.org/10.1038/s41586-021-04303-7
    nu = 1.54e-5;
    % Cuticle density (kg/m^3)
    % see https://doi.org/10.1038/s41586-021-04303-7
    rhow = 1200;
    % Young's modulus (Pa)
    % see https://doi.org/10.1016/j.isci.2021.103692
    Ew = 21e9;
    % Dimensionless geometrical radius of the 2nd moment of area
    R2hat = 0.63;
    % Maximum deflection tangent,
    % rounded up from 8.1 deg,
    % see https://doi.org/10.1038/s41586-021-04303-7)
    yhatmmax = deg2rad(10);
    % Gap Reynolds number based on
    % the velocity at R2 during power stroke
    ReG_ps = 0.52;
    % Bristles should bend by no greater
    % than eta=18% of the doubled gap size 2*G (P. placentis data)
    eta = 0.18;
    % Time fraction of the power strokes
    % in the full flapping cycle
    tau_ps = 0.63;

    % Dimensionless distance between the hinge and the
    % center of blade
    xihat = 0.2+0.4*sqrt(5*R2hat^2-1);

    % Maximum chord length (m)
    cmax = 2*(1-xihat)*R;

    % Dimensional geometrical radius of gyration (m)
    R2 = R2hat*R;

    % Mean velocity at the center of the plate
    Ups = 2*Phi*(f/tau_ps)*R2;

```

```
    % Average gap between bristles (m) is determined
    % from ReG constraint
    G = ReG_ps * nu / Ups;

    % Optimization parameter range for the
    % dimensionless bristle length
    lhat = (0.001:0.0001:(1-xihat)).';

    % Dimensionless radius of the membrane
    rhat = 1-xihat-lhat;

    % Aerodynamic force coefficient
    %cF = 2.0;
    cmean = (pi*(1-xihat)^2*R.^2) ./ R;
    cF = 15.20./(Ups.*cmean./nu) + 1.63;

    % Total aerodynamic force acting on the wing
    Ftot = cF*rho*Ups^2/2*pi*(1-xihat)^2*R^2;

    % Number of bristles, first approximation
    N_approx = round(2*pi*(rhat+lhat/2)*R / G);

    % Per meter length aerodynamic load of one bristle,
    % first aproximaton
    q_approx = Ftot ./ N_approx ./ (lhat*R) .* ...
        (1-((1-xihat-lhat)./(1-xihat)).^2);

    % Bristle diameter, first approximation
    D_approx = lhat.*(4*q_approx/(pi*Ew*eta*G)).^(1/4)*R;

    % Loop until convergence in the number of bristles
    converged = 0;
    iter = 0;
    while (~converged)

        % Next iteration
        iter = iter + 1;

        % Number of bristles, corrected
        N = round(2*pi*(rhat+lhat/2)*R ./ (G + D_approx));

        % Display iteration count
        if (iter > 10)
            disp(strcat("iter=",num2str(iter)));
        end

        % Per meter length aerodynamic load of one bristle,
        % corrected
        q = Ftot ./ N ./ (lhat*R) .* ...
            (1-((1-xihat-lhat)./(1-xihat)).^2);

        % Dimensional bristle diameter, corrected
        D = lhat.*(4*q/(pi*Ew*eta*G)).^(1/4)*R;

        % Check for convergence
        if (N == N_approx)
            converged = 1;
        end

        % Keep values from previous iteration
        D_approx = D;
```

```
        N_approx = N;
    end

    % Dimensionless radius of the bristle is determined
    % by bristle bending
    atilde = 0.5*D ./ (lhat*R);

    % Dimensionless membrane thickness is determined
    % from membrane bending
    hhatm = (4*Ftot/Ew/R^2 * ...
        xihat^3.*(xihat+rhat)/pi ./ ...
        rhat.^2/yhatmmax).^(1/3);

    % Wing mass
    mw = pi*rhow*R^3.*(hhatm .* ...
        (1-xihat-lhat).^2+N.*lhat.^3.*atilde.^2);

    % Wing moment of inertia with respect to the y axis
    Jw = zeros(size(mw));
    for jw = 1:length(Jw)
       Jw(jw) = func_moi(N(jw),R,xihat,lhat(jw), ...
           hhatm(jw),rhow,atilde(jw));
    end

    % Check for real values
    Jw(~isreal(Jw)) = Inf;

    % Exclude values that are impossible geometrically
    Jw((D.*N)./(2*pi*(1-xihat-lhat)*R)>1) = Inf;

    % Find minimum inertia design
    [Jw_opt(j),ind] = min(Jw);

    % Mass of the optimal design
    mw_opt(j) = mw(ind);

    % Optimal bristle length
    l_opt(j) = lhat(ind)*R;

    % Optimal bristle diameter
    D_opt(j) = D(ind);

    % Bristle spacing
    G_opt(j) = G;

    % Optimal number of bristles
    N_opt(j) = N(ind);

    % Optimal membrane thickness
    hhatm_opt(j) = hhatm(ind);

    % Total aerodynamic force
    Ftot_opt(j) = Ftot;
end

% Optimal membrane radius
r_opt = Rvec.*(1-xihat)-l_opt;

% Part of the area formed by setae
Amembrane = pi*r_opt.^2;
Awing = pi*(r_opt+l_opt).^2;
```

```
Abristles = Awing-Amembrane;
relative_setae_area = Abristles./Awing;
relative_membrane_area = Amembrane./Awing;

% Save workspace
save('wkspc_baseline.mat');

% Plot
h_fig = figure(1);
set(h_fig,'Units','centimeters', ...
    'Position',1.0*[15 2.4 8.4 7.0], ...
    'Resize','on','PaperPositionMode','auto');
clf;
loglog(Rvec*1e3,N_opt,'k.','LineWidth',1); hold on;
axis([0.1 5 1 10000]);
xlabel('$R$ (mm)','Interpreter','LaTeX');
ylabel('$N$ (-)','Interpreter','LaTeX');
grid on;

h_fig = figure(2);
set(h_fig,'Units','centimeters', ...
    'Position',1.0*[25 2.4 8.4 7.0], ...
    'Resize','on','PaperPositionMode','auto');
clf;
loglog(Rvec*1e3,G_opt*1e6,'g-','LineWidth',1); hold on;
loglog(Rvec*1e3,D_opt*1e6,'r-','LineWidth',1);
axis([0.1 5 0.1 100]);
xlabel('$R$ (mm)','Interpreter','LaTeX');
ylabel('$G$ ($\mu$m), $D$ ($\mu$m)','Interpreter','LaTeX');
grid on;
legend('$G$','$D$','Location','NorthEast', ...
    'Interpreter','LaTeX');
```

```
function [Jyy_tot] = func_moi(N,R,xihat,lhat,hhatm,rhow,atilde)
%func_moi.m : Moment of inertia about y-axis
% N : Number of bristles
% R : Wing length
% xihat: Relative centroid x coordinate
% lhat : Dimensionless bristles length
% hhatm : Dimensionless membrane thickness
% rhow : Material density in kg/m^3
% atilde : Bristle radius relative to the bristle length

% Wing centroid offset
xi = xihat*R;

% Membranous part radius
r = R*(1-xihat-lhat);

% Bristle length
l = R*lhat;

% Bristle orientations
th_vec = 2*pi*(0:N-1).'/N;

% Bristle center of mass coordinate
xbrcg = xi + (r+l/2)*cos(th_vec);

% Mass of a bristle
mb = pi*rhow*l^3*atilde^2;

% Bristle moment of inertia with respect to
```

```
% vertical (y) axis
Jyy_bristles = sum(mb*(l^2*cos(th_vec).^2+xbrcg.^2));

% Blade moment of inertia with respect to the y axis
Jyy_blade = pi*rhow*R^5*hhatm*(1-xihat-lhat)^2* ...
    (0.25*(1-xihat-lhat)^2+xihat^2);

% Total moment of inertia
Jyy_tot = Jyy_bristles + Jyy_blade;
end
```

6. Exploratory analysis of Mymaridae

Mymaridae are a family of tiny wasps that typically have bristled wings; therefore, they might be expected to follow the same optimality rules as Ptiliidae. The primary obstacle to quantitatively testing the optimality hypothesis is the lack of kinematic data. In the analysis that follows, we infer the values and trends of the necessary kinematic parameters from morphology and from available data on other Chalcidoidea. Another challenge arises from the greater diversity within Mymaridae, particularly in wing shapes and, most likely, in their kinematics. Consequently, the assumption of geometric similarity becomes less plausible. These limitations render the analysis presented in this section somewhat speculative; nonetheless, we hope it remains of value.

We performed morphometrics of the front wing in 62 species of Mymaridae using published images (11, 12) and measured the following parameters: wing length R; number of setae (bristles) N; dimensionless radius of the 2nd moment of area $\hat{R}_2$; part of wing area occupied by setae $(1 - A_{membrane})/A_{total} \times 100\%$ and average gap between bristles G (SI Dataset 2). For Mymaridae, the same scheme of morphometric measurements was used as for Ptiliidae (Fig. 2A in the main text).

An immediate contrasting result compared to the trends previously detailed for Ptiliidae is that the number of bristles exhibits an almost linear increase with R ($N \propto R^{0.97}$), and the relationship between bristle spacing and wing length is given by $G/R \propto R^{-0.88}$. If the gap-based Reynolds number Re_{Gps}, the fraction of power stroke τ_{ps} and the amplitude Φ are held constant, we obtain a relation for the the frequency

$$f = \frac{Re_{Gps} \nu \tau_{ps}}{2\Phi \hat{R}_2} \frac{1}{GR}. \qquad [12]$$

After substituting the allometry for G/R, we find $f = \propto R^{-1.12}$ which is close to the interspecific allometry trend $f = \propto R^{-1}$ suggested by Weis-Fogh (13). In the following analysis, we use the formula

$$f = 0.122\,\mathrm{Hz\,m} \times R^{-1}, \qquad [13]$$

which evaluates to, e.g., $f = 244$ Hz at $R = 0.5$ mm. This relation replaces the originally constant frequency in the optimization code. Of course, it requires further verification by direct measurement. If confirmed, a possible explanation may be the lack of geometrical similarity due to large morphological variability in Mymaridae, resulting in a trend close to the interspecific allometry. Then it may become necessary to subdivide Mymaridae in morphologically similar groups before applying the optimality analysis.

We estimated the flapping amplitude as $\Phi = 145.5\,\mathrm{deg}$ and the time fraction of the power stroke as $\tau_{ps} = 0.5751$ on the basis of data on *Encarsia formosa*, a membranous-winged wasp of the Aphelinidae family (1). Other parameters that differ from the original Ptiliidae values include the dimensionless radius of the 2nd moment of area, $\hat{R}_2 = 0.688$ (median value for Mymaridae, SI Dataset 2); the gap-based Reynolds number ($Re_{Gps} = 0.7$ estimated using (13) and the available morphological data). Finally, in the absence of wing deformation measurement, we hypothesize that Mymaridae wings can bend slightly more ($\hat{\gamma} = \arctan(20\,\mathrm{deg})$), but the bristles are much stiffer ($\eta = 1\%$). Unlike Ptiliidae, Mymaridae do not have elytra to protect their wings. The bristles in Mymaridae do not possess secondary outgrowths structures. Therefore, the stem diameter is determined by the requirements for wear resistance and aerodynamic efficiency, which may result in a diameter larger than what is necessary to resist bending.

The results are shown in Supplementary Figure S6 in comparison with Ptiliidae the optimization trends reported in the main text. The data point values for $A_{membrane}/A_{total}$ (Supplementary Figure S6A) are dispersed between 9% and 78% without any significant trend. This reflects the significant morphological variability and a lack of self-similarity in Mymaridae. The trend predicted by the model lies close to the lower bound of the data point cloud. A good alignment between model evaluations for G/R and N and the respective data points (Supplementary Figure S6B,C) is ensured by the prescribing $f(R)$ as explained above, see equation (13). Bristle diameter data are very limited. Our model suggests that D in Mymaridae must be generally larger than in Ptiliidae (Supplementary Figure S6D). The optimized wing mass, which is shown in Supplementary Figure S6E in the dimensional form, is smaller in Mymaridae only for wings that are longer than 0.8 mm. The slow decay in the limit of small R is explained by the frequency diverging as $1/R$. All these results may serve as guidance for identifying critical aspects when designing new experiments and conducting measurements.

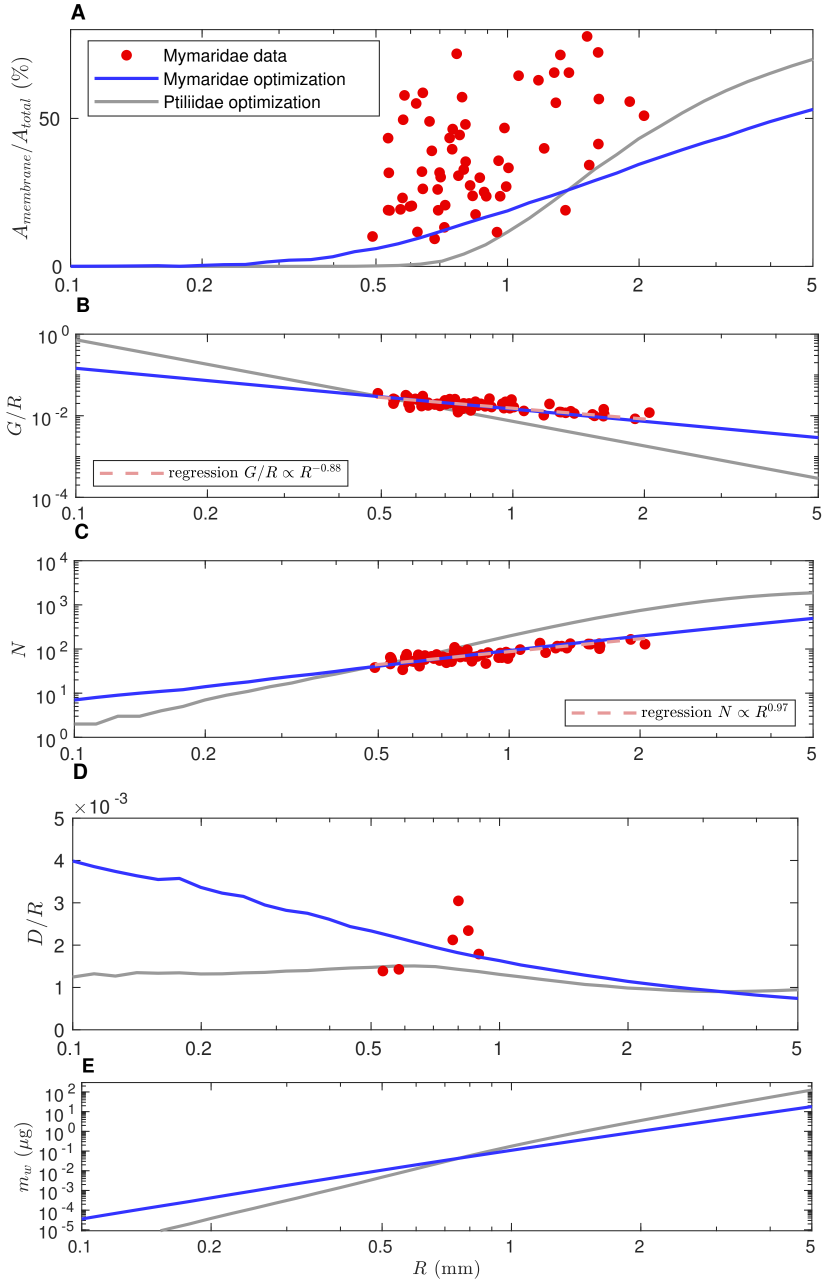

Fig. S6. Parameters of Mymaridae wings (in comparison with the Ptiliidae-based optimization results): (A) relative membranous surface area $A_{membrane}/A_{total}$; (B) relative gap G/R; (C) number of bristles N and (D) relative diameter D/R. (E) Mass of the optimal bristled wing m_w, in µg. Solid circles are data from morphological measurements in Mymaridae (from published sources); pale dashed lines are regression plots on the same data. Solid blue lines are the results of optimization for Mymaridae. Solid grey lines are the optimal plots for Ptiliidae, as in Fig. 3 of the main text.

SI Dataset S1 (**supporting_information_dataset1.xlsx**)

Ptiliidae morphometric, kinematic and wing bending data.

SI Dataset S2 (**supporting_information_dataset1.xlsx**)

Mymaridae morphometric data.

References

1. X Cheng, M Sun, Wing-kinematics measurement and aerodynamics in a small insect in hovering flight. *Sci. Reports* **6**, 25706 (2016).
2. X Cheng, M Sun, Very small insects use novel wing flapping and drag principle to generate the weight-supporting vertical force. *J. Fluid Mech.* **855**, 646–670 (2018).
3. SE Farisenkov, et al., Novel flight style and light wings boost flight performance of tiny beetles. *Nature* **602**, 96–100 (2022).
4. D Kolomenskiy, et al., The dynamics of passive feathering rotation in hovering flight of bumblebees. *J. Fluids Struct.* **91**, 102628 (2019).
5. Y Liu, M Sun, Wing kinematics measurement and aerodynamics of hovering droneflies. *J. Exp. Biol.* **211**, 2014–2025 (2008).
6. YZ Lyu, HJ Zhu, M Sun, Flapping-mode changes and aerodynamic mechanisms in miniature insects. *Phys. Rev. E* **99**, 012419 (2019).
7. XG Meng, M Sun, Aerodynamics and vortical structures in hovering fruitflies. *Phys. Fluids* **27** (2015) 031901.
8. S Oh, B Lee, H Park, H Choi, ST Kim, A numerical and theoretical study of the aerodynamic performance of a hovering rhinoceros beetle (Trypoxylus dichotomus). *J. Fluid Mech.* **885**, A18 (2020).
9. T Weis-Fogh, Quick estimates of flight fitness in hovering animals, including novel mechanisms for lift production. *J. Exp. Biol.* **59**, 169–230 (1973).
10. CP Ellington, The aerodynamics of hovering insect flight. II. Morphological parameters. *Philos. Transactions Royal Soc. London. B, Biol. Sci.* **305**, 17–40 (1984).
11. NQ Lin, JT Huber, J la Salle, The Australian genera of Mymaridae (Hymenoptera: Chalcidoidea). *Zootaxa* **1596**, 1–111 (2007).
12. JT Huber, JD Read, SV Triapitsyn, Illustrated key to genera and catalogue of Mymaridae (Hymenoptera) in America north of Mexico. *Zootaxa* **4773**, 1–411 (2020).
13. T Weis-Fogh, Dimensional analysis of hovering flight in *Scale Effects in Animal Locomotion: Based on the Proceedings of an International Symposium Held at Cambridge University, September, 1975*, ed. T Pedley. (Academic Press), p. 405 (1977).
